# Devisable POM/Ni Foam Composite: Precisely Control Synthesis toward Enhanced Hydrogen Evolution Reaction at High pH

**DOI:** 10.1002/chem.201903059

**Published:** 2019-11-04

**Authors:** Xueying Jia, Carsten Streb, Yu‐Fei Song

**Affiliations:** ^1^ State Key Laboratory of Chemical Resource Engineering Beijing University of Chemical Technology Beijing 100029 P. R. China; ^2^ Institute of Inorganic Chemistry I Ulm University Albert-Einstein-Allee 11 89081 Ulm Germany

**Keywords:** composites, electrocatalysis, hydrogen evolution reaction, polyoxometalates, self-assembly

## Abstract

Polyoxometalates (POMs) are promising catalysts for the electrochemical hydrogen production from water owing to their high intrinsic catalytic activity and chemical tunability. However, poor electrical conductivity and easy detachment of the POMs from the electrode cause significant challenges under operating condition. Herein, a simple one‐step hydrothermal method is reported to synthesize a series of Dexter–Silverton POM/Ni foam composites (denoted as Ni**M**‐POM/Ni; **M**=Co, Zn, Mn), in which the stable linkage between the POM catalysts and the Ni foam electrodes lead to high activity for the hydrogen evolution reaction (HER). Among them, the highest HER performance can be observed in the NiCo‐POM/Ni, featuring an overpotential of 64 mV (at 10 mA cm^−2^, vs. reversible hydrogen electrode), and a Tafel slope of 75 mV dec^−1^ in 1.0 m aqueous KOH. Moreover, the NiCo‐POM/Ni catalyst showed a high faradaic efficiency ≈97 % for HER. Post‐catalytic of NiCo‐POM/Ni analyses showed virtually no mechanical or chemical degradation. The findings propose a facile and inexpensive method to design stable and effective POM‐based catalysts for HER in alkaline water electrolysis.

## Graphical abstract

**HER evolution**: A series of Dexter–Silverton‐type Ni**M**‐POMs on Ni foam electrodes has been successfully deposited for HER in alkaline media. Among them, the NiCo‐POM/Ni exhibited the best performance with low overpotential of 64 mV and Tafel slope of 75 mV dec^−1^ at current density of 10 mA cm^‐2^.

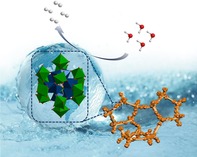

## Introduction

Hydrogen is an attractive sustainable energy carrier to address the challenges related with fossil fuel.^1^, ^2^ One of the most promising routes to generate hydrogen is electrochemical water splitting. The hydrogen evolution reaction (HER) reaction is a typical two‐electron transfer reaction, and involves interfacial proton‐coupled electron transfer and concomitant hydrogen evolution.^3^, ^4^, ^5^ Compared with the electrolysis process in acidic media, HER in alkaline electrolysis is more favourable for electrochemical water splitting due to the robustness of electrode materials, long lifetime of catalysts, cheap electrolyser construction and less equipment corrosion.^6^, ^7^, ^8^ However, HER reaction rate for most catalysts in alkaline solution is 2–3 orders of magnitude lower than that in acidic solution,^9^, ^10^ and HER application is largely limited by sluggish kinetics in alkaline solution. Therefore, significant studies are focusing on the development of electrocatalysts to overcome the high energy barriers and slow kinetics of HER reaction in alkaline solution. To date, the platinum‐based catalysts are still widely used as HER catalysts, which combine low overpotential with high operational stability.^11^, ^12^, ^13^, ^14^ However, the low abundance and high cost of Pt‐based electrocatalysts cause serious problems for widespread commercialization of HER technology.^15^ As such, academic and industrial communities spare no efforts in exploring efficient, stable and easily accessible HER catalysts based on earth‐abundant metals.

Polyoxometalates (POMs), as a class of anionic transition metal oxides, which show a rich diversity of structural types and reversible multi‐electron redox behaviours.^16^, ^17^, ^18^ Owing to their unique electron sponge behaviour, POMs exhibit proton‐coupled multi‐electron transfers.^19^, ^20^, ^21^, ^22^ More importantly, the electrocatalytic efficiency can be tuned by adjusting the chemical composition and microenvironment of POMs.^23^, ^24^, ^25^, ^26^ In terms of POMs application in HER, several POM‐based electrocatalysts for HER in acidic solution were reported so far. For example, Zhang et al. presented the P_8_W_48_/rGO nanomaterial as an efficient electrocatalyst for the HER in acidic aqueous solution.^27^ Ma et al. synthesized two POM‐encapsulated twenty‐nuclear silver‐tetrazole nanocage frameworks, which exhibited high activity for HER in 0.5 m H_2_SO_4_ aqueous solution.^28^ In contrast, few studies can be found on POM‐based electrocatalysts for HER in alkaline media. Under such circumstances, development of highly active and stable electrocatalysts for HER at high pH by technologically viable and scalable synthetic routes remains highly challenging.

In this paper, we have successfully constructed a series of mixed metal polyoxometalate microcrystals on commercial Ni foam electrodes for high‐performance HER in alkaline media. The Keggin‐type POM clusters act as building blocks to form new Dexter–Silverton‐type POM‐based materials of Ni**M**‐POM (**M**=Co, Zn, Mn). Mechanistic studies indicate that the stable electrical “wiring” between electrocatalyst and electrode, together with a high number of accessible reaction sites lead to excellent HER performance comparable with commercial Pt/C catalysts under alkaline condition.^29^, ^30^, ^31^

## Results and Discussion

**Figure 1:**
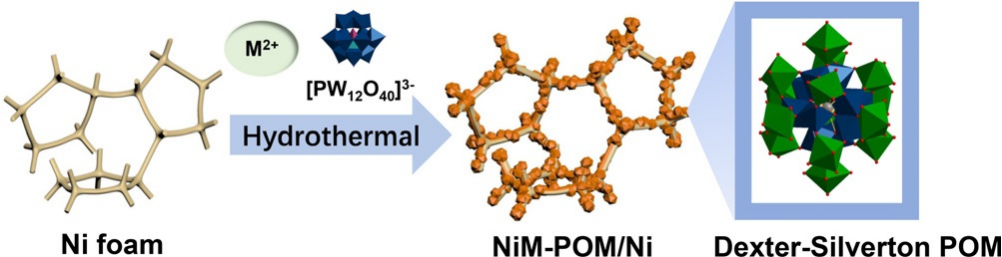
Left) Deposition of Dexter–Silverton polyoxometalate microcrystals on Ni foam electrode. Right) Structural representation of the Dexter–Silverton POM catalyst. W blue, Ni grey, **M** (**M**=Co, Zn, Mn) green, O red and hydrogen atoms omitted for clarity.

**Figure 2:**
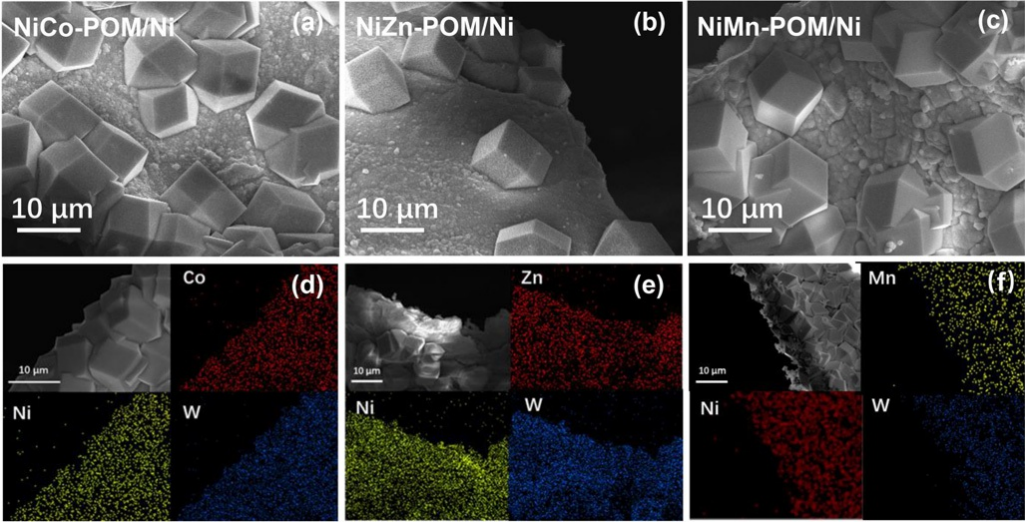
SEM images of a) NiCo‐POM/Ni, b) NiZn‐POM/Ni and c) NiMn‐POM/Ni electrode. EDX elemental mapping of d) NiCo‐POM/Ni electrode for the spatial distribution of Co, Ni and W. e) EDX elemental mapping of the NiZn‐POM/Ni electrode for the spatial distribution of Ni, Zn and W. f) EDX elemental mapping of the NiMn‐POM/Ni electrode for the spatial distribution of Ni, Mn and W.

The Ni**M**‐POM (**M**=Co, Zn, Mn) electrocatalysts was synthesized on commercial Ni foam electrodes by hydrothermal reaction, as illustrated in Figure 1. The reaction proceeded by mixing the Keggin‐type POM precursor [PW_12_O_40_]^3−^ and the respective transition metals of choice (Ni, Co, Mn, Zn) as nitrate salts in the presence of Ni foam under aqueous condition at 180 °C. Previous studies demonstrated that the Ni^2+^ ions were released from the Ni foam during hydrothermal synthesis and can be incorporated into the POM lattice. As a result, the Dexter–Silverton clusters (denoted as NiCo‐POM/Ni, NiZn‐POM/Ni and NiMn‐POM/Ni) can be obtained successfully as microcrystals (Figure 2).

Scanning electron microscopy images (Figure 2 a–c) revealed that the uniformly sized rhombic dodecahedral crystals of Ni**M**‐POM/Ni (**M**=Co, Zn, Mn) were homogeneously distributed onto the Ni foam. No mechanical detachment sites were observed. The corresponding energy‐dispersive X‐ray spectroscopic (EDX) elemental mapping (Figure 2 d–f) showed the homogeneous distribution of the respective transition metal (**M**=Co, Mn, Zn) and W, indicating the successful synthesis of Ni**M**‐POM/Ni.

**Figure 3:**
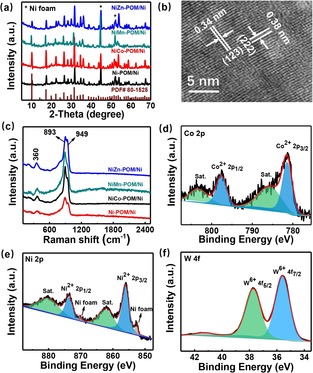
a) Powder XRD data for the Ni**M**‐POM/Ni electrode. b) HRTEM images of NiCo‐POM. c) Raman spectra of Ni**M**‐POM/Ni electrodes. High‐resolution XPS spectra of NiCo‐POM/Ni showing the d) Co2p, e) Ni2p and f) W4f regions.

The X‐ray diffraction (XRD) pattern of the Ni**M**‐POM/Ni (Figure 3 a) showed the standard diffraction patterns of the Dexter–Silverton POM of [M_8_W_12_O_42_(OH)_4_(H_2_O)_8_] (database no. ICDD‐PDF‐80–1525).^32^ Furthermore, a representative sample of NiCo‐POM/Ni was studied by high‐resolution transmission electron microscopy (HRTEM, Figure 3 b) and showed lattice fringes with interplanar distances of 0.34 and 0.38 nm, respectively, corresponding to the (1 2 3) and (2 2 2) planes of NiCo‐POM. Raman spectra of Ni**M**‐POM/Ni (Figure 3 c) provided further structural information. The signal at 949 cm^−1^ can be assigned to ν_sym_ (W=O) stretching, and the corresponding asymmetric stretching mode (ν_asym_ W=O) and bending mode (δ W=O) can be observed at 893 and 360 cm^−1^, respectively. FTIR analysis (Figure S2) confirmed the expected POM‐based M−O vibrations in the range of 600–1000 cm^−1^.^33^

To gain further insight into the surface chemical composition and valence states of the NiCo‐POM/Ni, X‐ray photoelectron spectroscopy (XPS) was performed. The normalized high resolution XPS spectra for the Co 2p, Ni 2p, and W 4f levels (using the C 1s peak at 284.8 eV as a reference) were presented in Figure 3 d–f, respectively. Two Co 2p_3/2_ and Co 2p_1/2_ peaks can be observed at 781.5 and 797.7 eV, respectively, corresponding to the oxidation state of Co^2+^.^34^ The high‐resolution Ni 2p XPS data revealed the presence of nickel in oxidation state Ni^0^ (870.2 and 852.9 eV) and Ni^2+^ (873.7 and 856.1 eV).^35^, ^36^ For the W 4f of NiCo‐POM/Ni (Figure 3 f), the main peaks at 37.7 and 35.6 eV were W^6+^ 4f_7/2_ and W^6+^ 4f_5/2_ components, respectively, and no peak of the reduced W^5+^ or W^4+^ was observed, indicating the fully oxidized nature of W^6+^. As shown in Figure S3, the deconvoluted O 1s XPS spectrum displayed three peaks due to the lattice O^2−^ (530.6 eV), equatorial OH^−^ (531.5 eV), and water molecules (533.5 eV).

**Figure 4:**
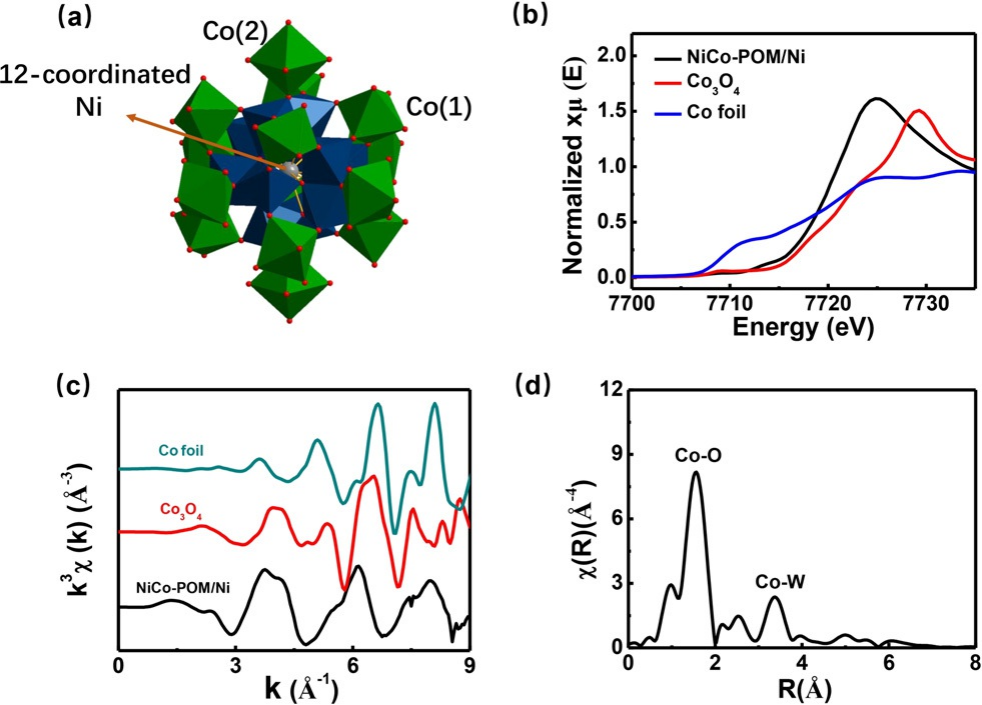
a) Structural representation of the Dexter–Silverton NiCo‐POM. Colour scheme: Ni grey, Co green, W blue, O red. Hydrogen atoms omitted for clarity. b) Co K‐edge XANES spectra. c) Co K‐edge extended XANES oscillation functions *k* ^3^ *χ*(*k*) for NiCo‐POM/Ni, Co_3_O_4_ and Co foil. d) Corresponding Fourier‐transformed Co R‐space spectrum of NiCo‐POM (distances are not corrected for photoelectron phase shifts.).

The accurate structure of the NiCo‐POM was further investigated by X‐ray absorption near edge structure (XANES). The Co K‐edge XANES spectra of NiCo‐POM/Ni, Co_3_O_4_ and Co foil were presented in Figure 4. By comparing the Co K‐edge of NiCo‐POM/Ni sample with that of Co and Co_3_O_4_, the NiCo‐POM/Ni exhibited a slight shift to lower energy, indicating the +2 oxidation state of Co in the NiCo‐POM.^37^, ^38^ It was consistent with that of XPS results. The Co K‐edge *k* ^3^ *χ*(*k*) oscillation curve (Figure 4 c) displayed that the oscillation amplitude of NiCo‐POM/Ni was smaller than those observed in the Co_3_O_4_ and Co foil, indicating structural difference in the coordination environment surrounding the Co atoms. As shown in Figure 4 d, the corresponding Fourier transformed R‐space spectra of NiCo‐POM/Ni exhibited two peaks at ≈1.6 and ≈3.3 Å. The peak at 1.6 Å corresponded to the first octahedral coordination of Co(1)−O shell, while the peak at 3.3 Å was attributed to the scattering between the Co and the W in the second nearest neighbour shell. Notably, a weak peak at 2.6 Å was assigned to Co(2) coordination with OH anions (bond distance 3.08 Å) (Figure 4 a).^32^, ^39^, ^40^ Furthermore, the XANES fitting of Dexter–Silverton NiCo‐POM/Ni indicated that the Co centres were six‐coordinate and occupied the nearly octahedral environment in the NiCo‐POM/Ni. Based on the XPS, XANES and ICP‐AES data (Table S1 in Supporting Information), a formula [Co_7_NiW_12_O_42_(OH)_4_(H_2_O)_8_] can be determined. The Ni^2+^ ions occupied the centre of [NiW_12_O_42_]^10−^ polyanion formed by six equivalent sets of two face‐sharing WO_6_ octahedral units {W_2_O_9_}.

**Figure 5:**
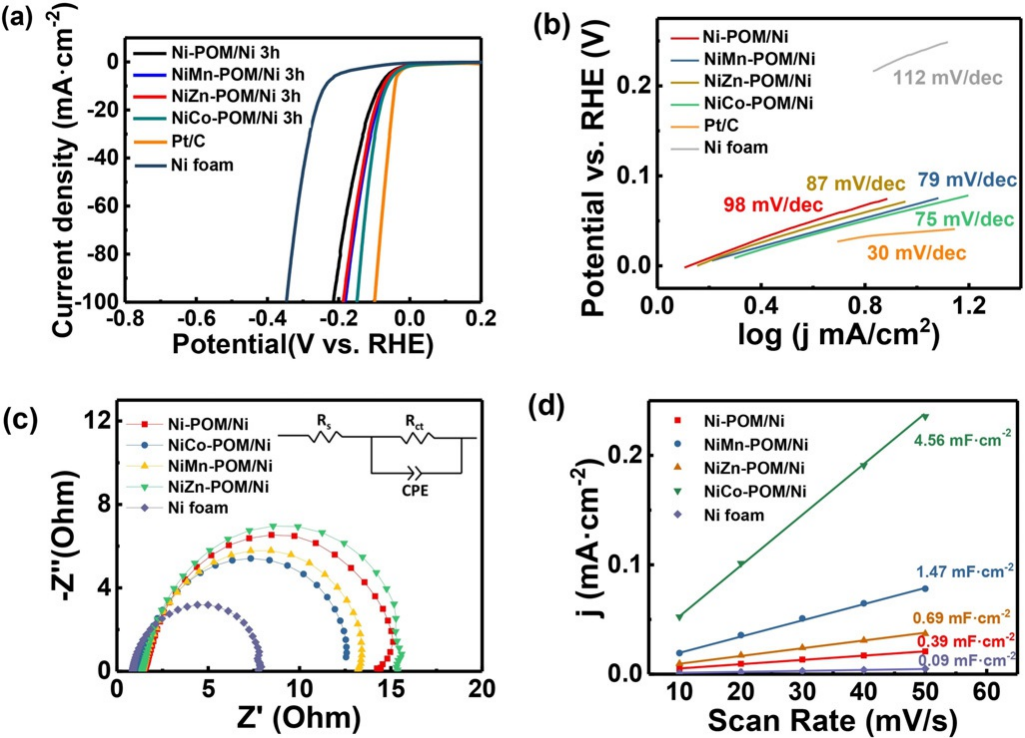
a) Polarization curves of Ni**M**‐POM/Ni electrode, commercial Pt/C and Ni foam in 1.0 m KOH at a scan rate of 5 mV s^−1^. b) Tafel plots of Ni**M**‐POM/Ni electrode and Ni foam. c) Nyquist plots of the Ni**M**‐POM/Ni and Ni foam recorded at selected overpotentials in an N_2_‐saturated 1.0 m KOH solution. d) Determination of the double‐layer capacitance *C*_dl_ of Ni**M**‐POM/Ni and Ni foam.

The Ni**M**‐POM/Ni composites were investigated as electrocatalysts for HER in alkaline aqueous KOH as electrolyte. In order to evaluate the HER performance of the Ni‐POM/Ni, NiCo‐POM/Ni, NiZn‐POM/Ni, and NiMn‐POM/Ni, a typical three‐electrode system in an N_2_‐saturated 1.0 m KOH aqueous solution was applied by using the corresponding composites as working electrodes, a saturated calomel electrode as reference electrode and a carbon rod as counter electrode. All potentials were referenced to the reversible hydrogen electrode (RHE), and the data were corrected for internal resistances (IR). Linear sweep voltammetry (LSV) of the composites (Figure 5 a) showed that all Ni**M**‐POM/Ni composites exhibited excellent electrocatalytic HER performance. Among them, the NiCo‐POM/Ni exhibited the best performance with a low onset potential of 8 mV as well as a low overpotential of 64 mV at a current density *j*=10 mA cm^−2^. In contrast, other composites showed slightly higher overpotential of 68 mV for NiMn‐POM/Ni, 74 mV for NiZn‐POM/Ni, and 83 mV for Ni‐POM/Ni. The overpotential of the Ni**M**‐POM/Ni electrodes are comparable to that of most Ni‐based materials for HER in alkaline solution (Table 1).

**Table 1:** Comparison of HER‐catalytic performance of Ni‐based electrocatalysts at the current density of 10 mA cm^−2^ in 1.0 KOH.

Material^[a]^	Overpotential [mV]	Tafel slope [mV dec^−1^]	Ref.
Ni_3_S_2_@NiV‐LDH/NF	126	90	46
(Ni,Co)Se_2_‐GA	128	79	47
Ni‐Mo_2_C/C	99	73	48
V‐Ni_2_P NSAs/CC	85	95	49
Ni_1−*x*_Co_*x*_Se_2_MNSN/NF	85	52	50
Ni_3_Fe@N‐CNT/NFs	72	98	51
Ni/Mo_2_C‐NCNFs	143	57.8	52
Se‐(NiCo)S_x_/(OH)_*x*_	103	87.3	53
Ni(OH)_2_@CuS	95	104	54
**NiCo‐POM/Ni**	**64**	**75**	**this work**

[a] NF: nickel foam, GA: 3D graphene aerogel, CC: carbon cloth; CNT: carbon nanotube; NCNFs: Nitrogen‐doped carbon nanofibers.

Figure 5 b displayed the Tafel plots of Ni**M**‐POM/Ni electrocatalysts, which provided deep insight into the HER reaction pathway on the electrocatalyst. The NiCo‐POM/Ni composite electrode possessed a Tafel slope of 75 mV dec^−1^, which was lower than that of NiMn‐POM/Ni (79 mV dec^−1^), NiZn‐POM/Ni (87 mV dec^−1^), and Ni‐POM/Ni (98 mV dec^−1^), respectively. These results suggested that the hydrogen evolution process followed the Volmer–Heyrovsky mechanism and the rate‐determining step was the hydrogen generation.^41^ NiCo‐POM/Ni composite exhibited relatively excellent performance may attribute to the presence of Co sites to provide more accessible H adsorption sites.^42^ Potentiostatic cathodic electrolysis was measured by maintaining NiCo‐POM/Ni electrode at the overpotential of 200 mV versus RHE for 60 min. The NiCo‐POM/Ni electrode exhibited ≈97 % faradaic efficiency for HER (Figure S6), suggesting a high selectivity to hydrogen with minimal faradaic losses.^43^, ^44^

To have a better understanding of the catalytic behaviour and internal resistance of the composite electrodes, we performed electrochemical impedance spectroscopy (EIS) for Ni**M**‐POM/Ni in 1.0 m KOH solution. The Nyquist plots (Figure 5 c) indicated the expected semicircular features associated with charge transfer resistances in the HER process. As revealed by the charge transfer resistance (*R*_ct_) in Table 2, the NiCo‐POM/Ni electrode displayed the *R*_ct_ of 11.9 Ω, which was lower than those of NiMn‐POM/Ni (12.6 Ω), NiZn‐POM/Ni (15.1 Ω) and Ni‐POM/Ni (13.6 Ω). This result indicated that the NiCo‐POM/Ni possessed the lowest charge‐transfer resistance and superior conductivity, which enabled efficient interfacial electron transport without a binder or conductive additive for HER.^45^ To rationalize whether this finding was correlated to the electrochemically active surface areas (ECSA) of the electrodes, the ECSA values was determined based on the double‐layer capacitance (*C*_dl_) calculated from voltammetric data in the non‐faradaic region. As shown in Figure 5 d, the NiCo‐POM/Ni showed the largest *C*_dl_ of 4.56 mF cm^−2^, while the *C*_dl_ of NiMn‐POM/Ni (1.47 mF cm^−2^) and NiZn‐POM/Ni (0.69 mF cm^−2^) were higher when compared with the monometallic Ni‐POM/Ni (0.39 mF cm^−2^) and Ni foam (0.09 mF cm^−2^). The high *C*_dl_ of NiCo‐POM/Ni was related to the enhanced ECSA (Table 2), indicating the increase of the accessible catalytic sites for HER. Therefore, the highest ECSA of NiCo‐POM/Ni can be beneficial to water adsorption.

**Table 2:** Comparison of the charge transfer resistance and ECSA of the NiM‐POM/Ni electrodes and Ni foam in 1.0 KOH solution.

Sample	*R*_ct_ [Ω]	ECSA [cm^2^]
Ni‐POM/Ni	13.6	10
NiCo‐POM/Ni	11.9	114
NiMn‐POM/Ni	12.6	37
NiZn‐POM/Ni	15.1	17
Ni foam	7.3	2

**Figure 6:**
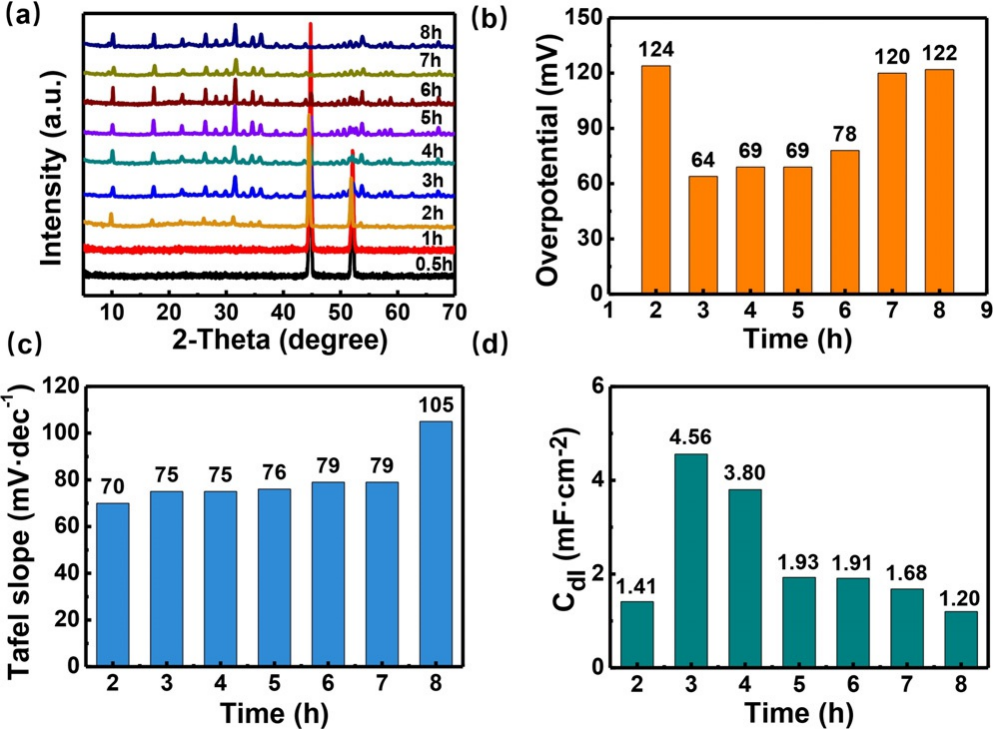
a) Powder XRD data for the NiCo‐POM/Ni electrodes obtained at different time. b) Change of overpotential (at *j*=10 mA cm^−2^) with NiCo‐POM particle size formed at different reaction time. c) Corresponding Tafel plots of NiCo‐POM obtained at different reaction time. d) Double layer capacitance *C*_dl_ of NiCo‐POM/Ni electrodes obtained at different reaction time.

Tuning the particle size of the POM microcrystals can be critical for controlling the number of surface‐accessible HER reaction sites.^55^ Taking the NiCo‐POM/Ni as an example, we examined the effect of average crystal size on HER activity. The NiCo‐POM microcrystal particle size can be easily controlled by variation of the hydrothermal reaction time. Particle formation can be monitored using powder XRD (Figure 6 a), where formation of the cubic NiCo‐POM phase started at reaction time of 2 hours. The size growth of NiCo‐POM on Ni foam can be observed up to maximum reaction time of 8 hours and no impurities were observed in the XRD. SEM images (Figure S1) of the NiCo‐POM/Ni composite obtained at different reaction time clearly revealed the growth process of the NiCo‐POM microcrystals on the Ni foam surface.

HER electrocatalysis studies (Figure 6 b) of these composites showed that the catalytic performance was related to the microcrystal size. The lower HER overpotentials (64–78 mV) were obtained for NiCo‐POM/Ni electrodes with average particle size of 2–4 μm at reaction time 3–6 h, while significant increase in overpotential can be found for microcrystal size with smaller than 1 μm and/or larger than 5 μm particle size. In addition, the NiCo‐POM/Ni with large particle‐size revealed increased Tafel slope, suggesting a kinetically favoured HER processes for the smaller particles (Figure 6 c). Further studies were carried out by examining the electrochemical double‐layer capacitance (*C*_dl_) (Figure 6 d). Herein, the NiCo‐POM/Ni with 2 μm average size exhibited the largest *C*_dl_, indicating that the NiCo‐POM/Ni featured the highest number of electrochemically active surface sites. It was further supported by EIS analyses (Figure S7). The NiCo‐POM/Ni with 2 μm particle size exhibited the lowest charge‐ transfer resistance, leading to efficient electron transport at the NiCo‐POM/Ni interface.^56^

**Figure 7:**
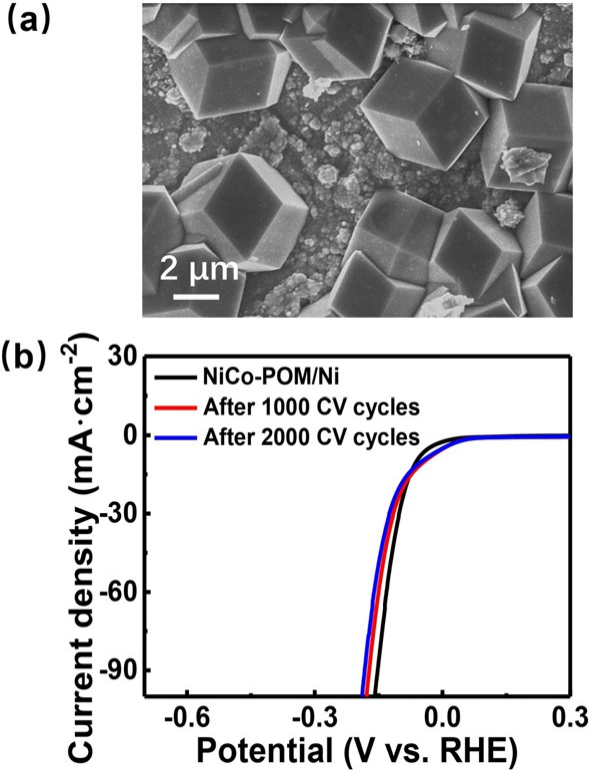
a) SEM image of NiCo‐POM/Ni electrode after HER. b) LSV polarization curves of NiCo‐POM/Ni after 0, 1000 and 2000 cycles in 1.0 m KOH.

The long‐term stability and degradation resistance of the NiCo‐POM/Ni electrode (particle size 2 μm, reaction time 3 h) were assessed by CV cycling at scan rate 5 mV s^−1^ in 1.0 m KOH. Figure 7 b showed three LSV curves of the electrode after 0, 1000 and 2000 cycles. It was found that almost negligible decrease highlighted the long‐term stability of the NiCo‐POM/Ni electrode. A post‐catalytic SEM image (Figure 7 a) indicated that the morphology of the NiCo‐POM catalyst was mostly retained, and no mechanical detachment of the catalyst from the electrode was observed. In addition, powder XRD analysis (Figure S8 a) and FTIR spectra (Figure S8 b) of the post‐catalytic electrode provided further support for the remarkable stability of the NiCo‐POM/Ni composite during sustained HER process in alkaline condition.

## Conclusions

In conclusion, a series of Ni**M**‐POM clusters were successfully fabricated on the porous Ni foam by hydrothermal methods. The as‐prepared Ni**M**‐POM/Ni composites showed outstanding reactivity and stability for HER in alkaline aqueous solution. Among them, the NiCo‐POM/Ni exhibited the best performance with low overpotential of 64 mV together with Tafel slope of 75 mV dec^−1^ at 10 mA cm^−2^. Besides, the chemically stable deposition of NiCo‐POM on the Ni foam led to their high reactivity and long‐term stability. Such excellent HER performance of the NiCo‐POM/Ni can be due to the intrinsic reactivity of POM microcrystals with abundant activity sites. To the best of our knowledge, the NiCo‐POM/Ni composite outperforms the most known Ni‐based HER electrocatalysts in alkaline media. Further work will expand this study to related systems to enable access to a new class of mixed‐metal oxides for energy‐relevant high‐performance electrocatalysts.

## Experimental Section

**Materials and Characterization**: All chemicals were of analytical grade and were used as received without any further purification. Na_3_[PW_12_O_40_]**⋅**6 H_2_O was synthesized according to a literature method.^57^

Fourier transform infrared (FTIR) spectra were recorded on a Bruker Vector 22 infrared spectrometer by using the KBr pellet method. Scanning electron microscopy (SEM) images were obtained by using a Zeiss Supra 55 SEM. The powder X‐ray diffraction (XRD) analysis was carried out on a Bruker D8 diffractometer with high‐intensity Cu${}_{K{}_{\alpha }}$ radiation. High‐resolution transmission electron microscopy (HRTEM) was conducted on a JEOL JEM‐2100 equipment under an accelerating voltage of 400 kV. The XPS spectra were acquired with PHI Quantera SXM Al with an monochromatized Al cathode (h*ν*=1486.6 eV) as the X‐ray source set at 100 W and a pass energy of 26.00 eV. Wide and detailed spectra were collected at 0° take‐off angle, using fixed analyser transmission mode with channel widths of 1.0 and 0.1 eV, respectively. The binding energies were calibrated based on C 1s (284.8 eV). Raman spectroscopy was performed on a Renishaw Raman spectrometer at a laser excitation wavelength of 532 nm. Inductively coupled plasma‐atomic emission spectroscopy (ICP‐AES) analysis was performed using a Shimadzu ICPS‐7500 spectrometer. X‐ray absorption near edge structure (XANES) measurements were obtained from the 1W1B beamline of the Beijing Synchrotron Radiation Facility (BSRF). The EXAFS data were processed and fitted in R‐space according to the standard procedures using the ATHENA and ARTEMIS modules implemented in the IFEFFIT software packages.

**Synthesis of the NiCo‐POM/Ni electrode**: The POM‐based electrode material was prepared by a simple hydrothermal process. The commercial Ni foam was cut into a 1×4 cm block, washed with acetone, aqueous HCl solution (2.0 m), deionized water and ethanol. 0.29 g Co(NO_3_)_2_ **⋅**6 H_2_O and 3.18 g Na_3_[PW_12_O_40_]**⋅**6 H_2_O were dissolved in 40 mL deionized water in a 50 mL Teflon autoclave. Then Ni foam was immersed in above solution and the reaction was heated to 180 °C for 3 hours to give the NiCo‐POM/Ni electrode.

**Electrochemical Measurements**: Electrochemical measurements were performed with an Instrument CHI 660E in 1.0 m aqueous KOH solution at room temperature in three‐electrode setup (working electrode: Ni**M**‐POM/Ni, Reference electrode: standard calomel electrode (SCE), counter electrode: carbon rod). Linear sweep voltammetry was performed with a scan rate of 5 mV s^−1^. EIS measurements were carried out in 1.0 m aqueous KOH at different potentials in the frequency range 0.1 to 105 Hz with an amplitude of 10 mV. The potential versus SCE was converted to the reversible hydrogen electrode (RHE) via the Nernst equation as follows: *E*_RHE_=*E*_SCE_+0.059 pH+*E* ^θ^_SCE_. The charge flow and hydrogen evolution were measured, and theoretical and experimental molar amounts of hydrogen evolution were calculated by Equations (1), (2), (3):

(1) $$n_{H_{2}}\left(theoretical\right)=\frac{Q}{2F}$$

(2) $$n_{H_{2\;}}\left(experimental\right)=\frac{PV}{RT}=\frac{PV\%(V_{cell}-V_{electrolyte})}{RT}$$

(3) $$\eta =\frac{n_{H_{2}}\left(experimental\right)+c_{\left(solu\;H_{2}\right)}V_{electrolyte}}{n_{H_{2}}\left(theoretical\right)}\times 100\%$$

Where $n_{H_{2}}\left(theoretical\right)$ is the theoretical number of moles of H_2_ produced, *Q* is the charge passed through the electrodes, *F* is the Faradaic constant (96485 C mol^−1^). The evolved amounts of H_2_ $n_{H_{2\;}}\left(experimental\right)$ were obtained by a water–gas displacing method, in which the H_2_ volume fraction (*V* %) of the hydrogen was analysed by gas chromatography (GC, Shimadzu GC‐2014C). *V*_cell_ is the initial volume of electrolyte, *V*_electrolyte_ is the residual volume of electrolyte in the electrolyser. *P* is the atmospheric pressure (105 kPa), *T* is the temperature (298 K), and *R* is the molar gas constant (8.314 J mol^−1^ K^−1^). And *c* ${}_{\left(\mathrm{solu}\;H{}_{2}\right)}$ is the solubility of H_2_ in water.

## Conflict of interest

The authors declare no conflict of interest.

## Supporting information

As a service to our authors and readers, this journal provides supporting information supplied by the authors. Such materials are peer reviewed and may be re‐organized for online delivery, but are not copy‐edited or typeset. Technical support issues arising from supporting information (other than missing files) should be addressed to the authors.

Supplementary
