# Supplementary material for: Devisable POM/Ni Foam Composite: Precisely Control Synthesis toward Enhanced Hydrogen Evolution Reaction at High pH
Source: Chemistry. 2019 Nov 4;25(68):15548–54. doi: 10.1002/chem.201903059 (PMC6973057; doi:10.1002/chem.201903059)
Supplement: Supplementary file 1 — Supplementary [file CHEM-25-15548-s001.pdf]

CHEMISTRY
A European Journal

Supporting Information

Devisable POM/Ni Foam Composite: Precisely Control Synthesis toward Enhanced Hydrogen Evolution Reaction at High pH

Xueying Jia,[a] Carsten Streb,*[b] and Yu-Fei Song*[a]

chem_201903059_sm_miscellaneous_information.pdf

Supporting Information

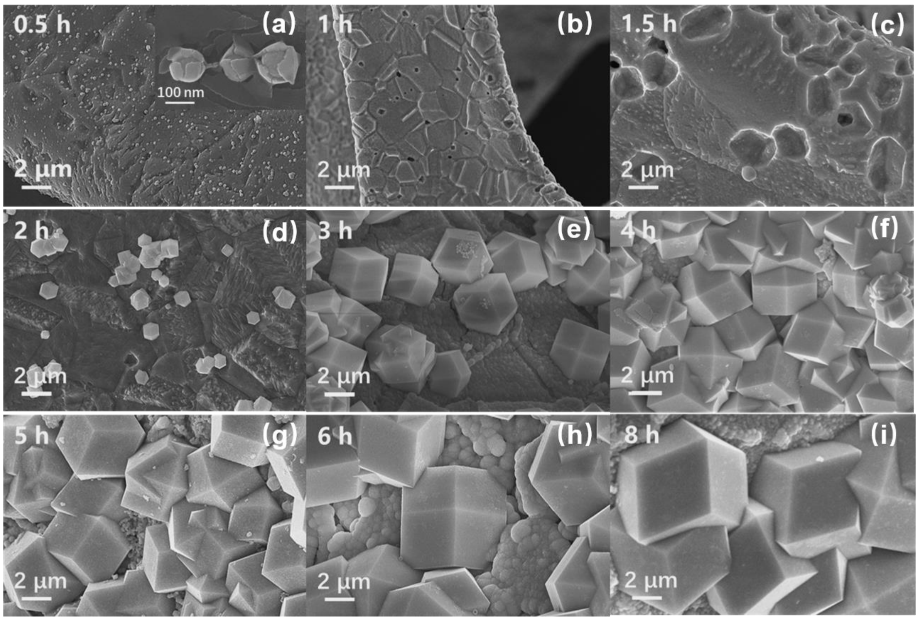

Figure S1. SEM images of NiCo-POM/Ni obtained at different reaction time.

Table S1. Metal content quantification of NiCo-POM by ICP-AES (wt.-%)

	Co	Ni	W
Found	11.82%	2.31%	65.73%
Calcd.	11.72%	2.06%	64.56%

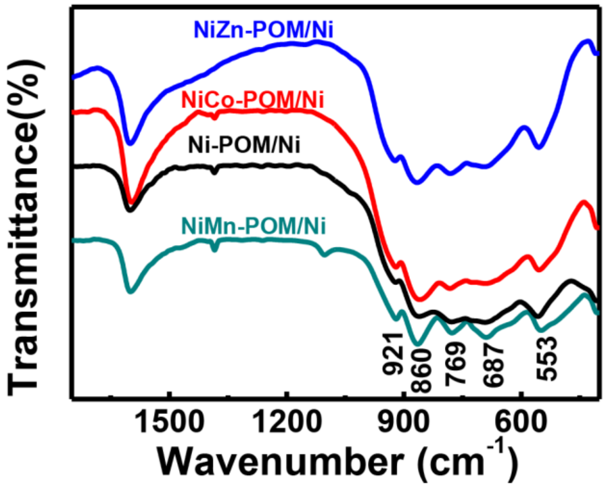

Figure S2. FT-IR spectra of Ni**M**-POM/Ni electrodes.

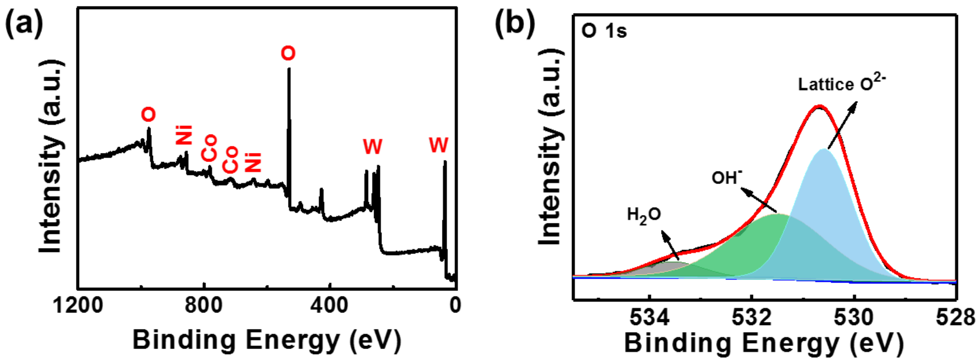

Figure S3. (a) XPS survey spectrum of NiCo-POM/Ni. (b) High-resolution XPS scan of NiCo-POM/Ni showing the O1s region.

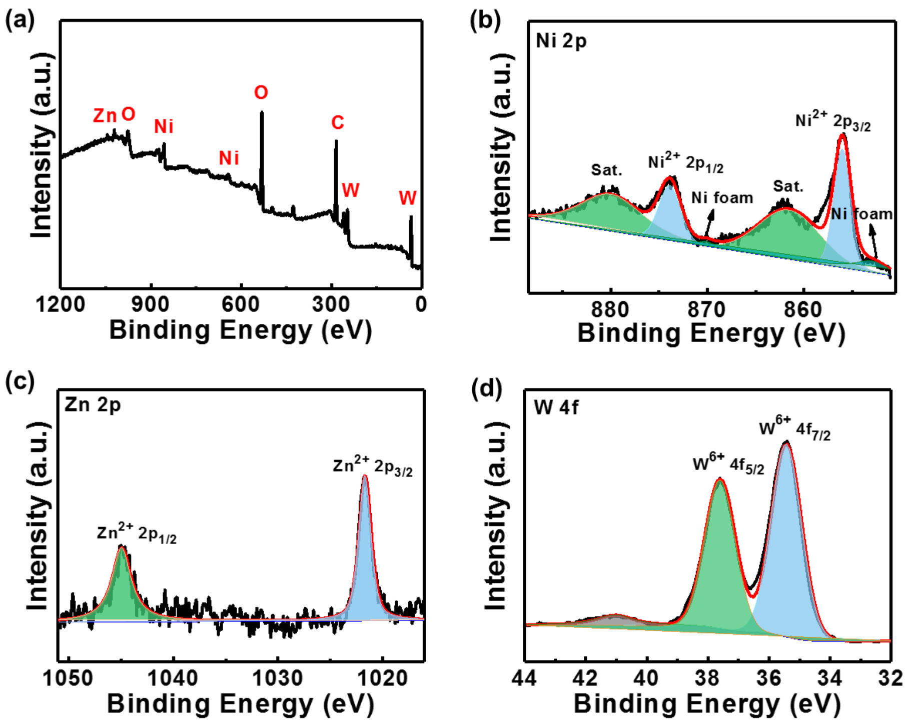

Figure S4. (a) XPS survey scan of the NiZn-POM/Ni sample. The high resolution XPS scans for (b) Ni 2p, (c) Zn 2p, (d) W 4f levels.

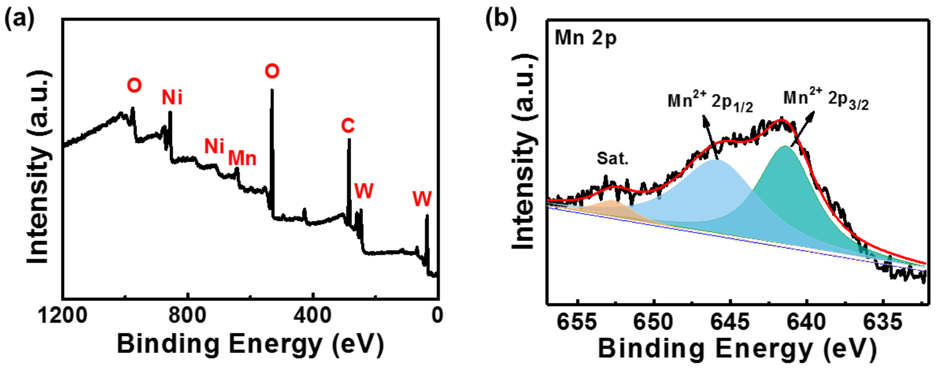

Figure S5. (a) XPS survey scan of the NiMn-POM/Ni sample; (b) The high resolution XPS scans for Mn 2p levels.

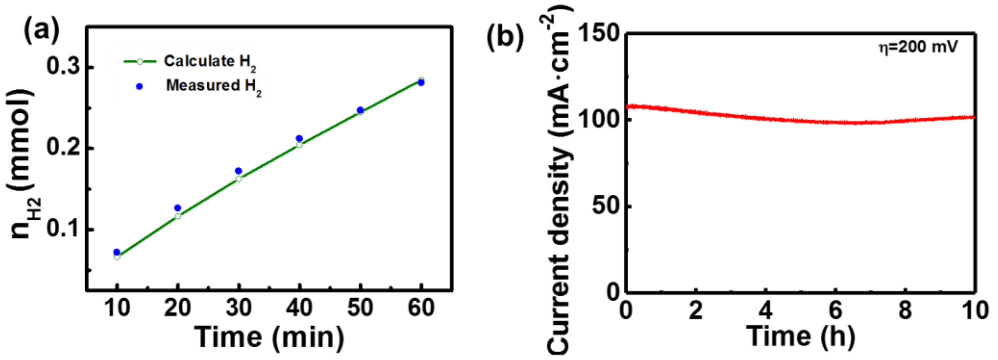

Figure S6. (a) The amount of gas theoretically calculated and experimentally measured versus time for HER of NiCo-POM/Ni. (b) Current density curve of NiCo-POM/Ni at the overpotential of 200 mV vs. RHE for 10h.

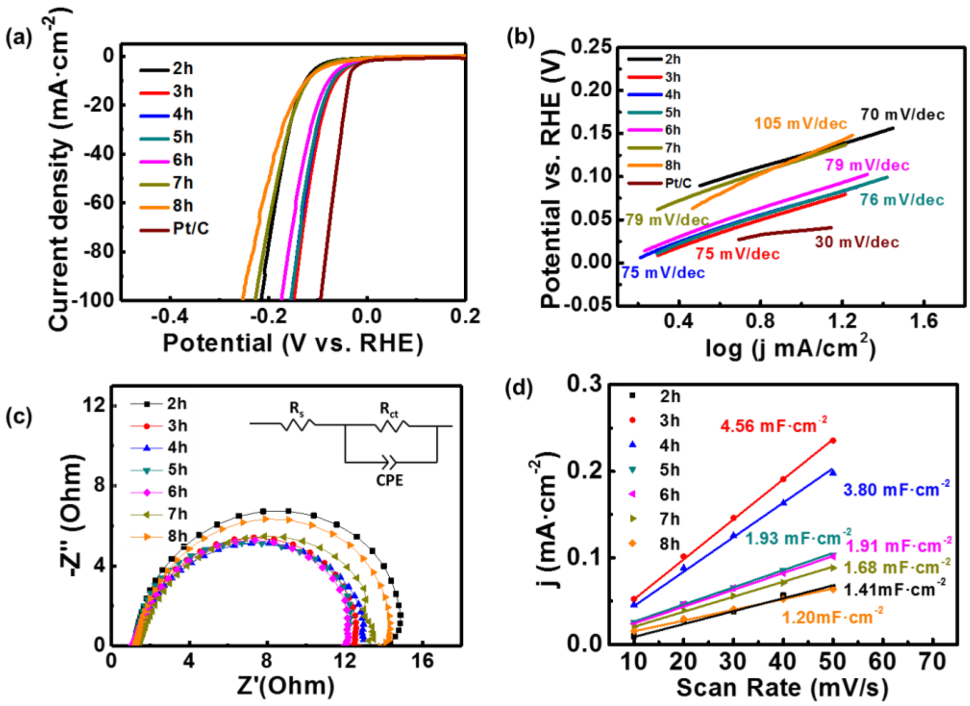

Figure S7. (a) IR-corrected polarization curves of NiCo-POM/Ni electrode obtained at different reaction time in aqueous 1.0 M KOH. (b) Tafel plots (c) Nyquist plots and (d) Plots used for evaluating the C_{dl} of the NiCo-POM/Ni obtained at different reaction time.

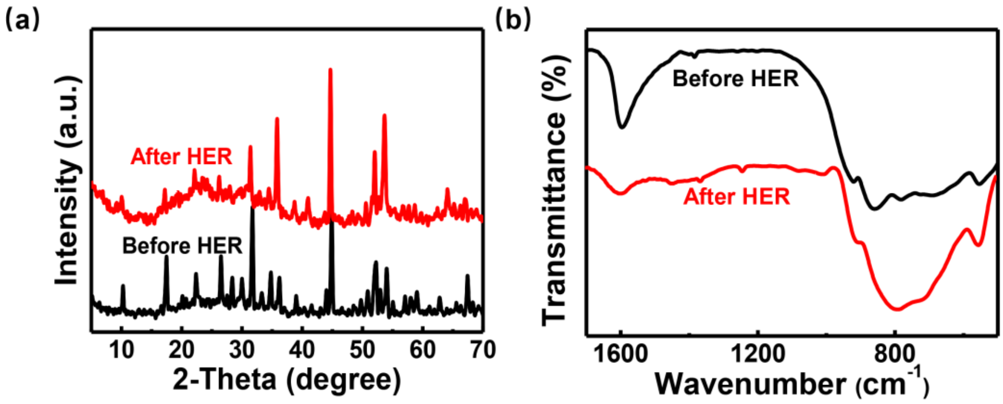

Figure S8. (a) Powder XRD data of NiCo-POM/Ni electrode before and after HER. (b) FT-IR spectra of NiCo-POM/Ni electrode before and after HER.

Note: After 2000 CV cycles, the recycled sample was directly used for XRD and FT-IR measurement as shown in Figure S8. XRD spectrum of the recycled sample showed different intensity in a few diffractions, and FT-IR spectrum of the recycled sample exhibited slight shift of the W-O stretching band. Such spectra changes can be attributed to the presence of various hydrogenated products (that cause the intensity change in the XRD) and adsorbed water (that cause the slight shift of W-O stretching bands in the FT-IR spectrum) in the recycled sample after HER measurement. It is likely that "the hydride could be formed by HER intermediates or a reaction between metallic Ni and dissolved hydrogen in the electrolyte" during HER catalytic reaction in alkaline solution of KOH. Moreover, ICP measurement of the KOH solution after the HER catalytic reaction showed that no detectable Ni and Co can be found, indicating good stability of NiCo-POM/Ni composite.
